# Effects of impairment in activities of daily living on predicting mortality following hip fracture surgery in studies using administrative healthcare databases

**DOI:** 10.1186/1471-2318-14-9

**Published:** 2014-01-28

**Authors:** Dallas P Seitz, Geoffrey M Anderson, Peter C Austin, Andrea Gruneir, Sudeep S Gill, Chaim M Bell, Paula A Rochon

**Affiliations:** 1 Department of Psychiatry, Queen’s University, Kingston, Ontario, Canada; 2 Institute of Health Policy, Management and Evaluation, University of Toronto, Toronto, Ontario, Canada; 3 Institute for Clinical Evaluative Sciences, Toronto, Ontario, Canada; 4 Women’s College Research Institute, Women’s College Hospital, Toronto, Ontario, Canada; 5 Department of Medicine, Queen’s University, Kingston, Ontario, Canada; 6 Institute for Clinical Evaluative Sciences, Queen’s University, Kingston, Ontario, Canada; 7 Department of Medicine, Mt. Sinai Hospital, Toronto, Ontario, Canada

**Keywords:** Hip fracture, Predictive models, Mortality, Outcomes, Activities of daily living, Confounding, Risk adjustment

## Abstract

**Background:**

Impairment in activities of daily living (ADL) is an important predictor of outcomes although many administrative databases lack information on ADL function. We evaluated the impact of ADL function on predicting postoperative mortality among older adults with hip fractures in Ontario, Canada.

**Methods:**

Sociodemographic and medical correlates of ADL impairment were first identified in a population of older adults with hip fractures who had ADL information available prior to hip fracture. A logistic regression model was developed to predict 360-day postoperative mortality and the predictive ability of this model were compared when ADL impairment was included or omitted from the model.

**Results:**

The study sample (N = 1,329) had a mean age of 85.2 years, were 72.8% female and the majority resided in long-term care (78.5%). Overall, 36.4% of individuals died within 360 days of surgery. After controlling for age, sex, medical comorbidity and medical conditions correlated with ADL impairment, addition of ADL measures improved the logistic regression model for predicting 360 day mortality (AIC = 1706.9 vs. 1695.0; c -statistic = 0.65 vs 0.67; difference in - 2 log likelihood ratios: χ2 = 16.9, p = 0.002).

**Conclusions:**

Direct measures of ADL impairment provides additional prognostic information on mortality for older adults with hip fractures even after controlling for medical comorbidity. Observational studies using administrative databases without measures of ADLs may be potentially prone to confounding and bias and case-mix adjustment for hip fracture outcomes should include ADL measures where these are available.

## Background

Several disease processes can lead to functional impairments in basic activities of daily living (ADLs) [1] and impairments in ADLs are common among older adults living in community [2-4] and long-term care (LTC) [5,6] settings. Measures of ADL impairment are important in observational studies as ADL may be a strong predictor of outcomes such as mortality [7-11] and long-term care (LTC) placement [12-15] and may act as a confounder if ADL impairment is imbalanced between comparison groups [16]. This potential confounding may be particularly problematic in observational studies of older adults with complex chronic diseases given that ADL impairment may be a stronger predictor of some outcomes such as LTC placement or mortality than some commonly used measures of medical comorbidity [17-19].

Comorbidity indices, such as the Charlson comorbidity score [20,21], the Johns Hopkins Adjusted Clinical Groups (ACG) system [22], or measures based on prescription medications [23], can be constructed from information in many administrative databases to risk adjust outcomes and control for confounding. However, these databases and comorbidity measures do not contain direct information on ADL impairment and as such residual confounding due to ADL impairment may be present in these studies. To date, there have been only a few studies which have evaluated the impact of ADL impairment on predicting outcomes in administrative databases [19,24,25] and there is limited information available on strategies to minimize the potential bias that may result when direct measures of ADL impairment are not available.

One potential method to control for confounding due to ADL impairment is to control for variables that are available in most administrative databases such as demographics, medical conditions [1,26-28], and measures of medical comorbidity which are correlated with ADL impairment. These proxy measures of ADL impairment may be more widely available in administrative data and may be able to minimize the effect of omitting information on direct measures of ADL impairment when such measures are not available. The goal of our study was to identify correlates of ADL impairment among older adults with hip fractures and to assess the impact of direct measures of ADL impairment on predicting mortality in the 360 days following hip fracture surgery.

## Methods

### Systematic review of factors associated with functional impairment

#### Electronic search strategy

To identify medical conditions and other correlates of functional impairment we searched electronic databases using key words and medical subject headings (Additional file 1). The databases Medline and EMBASE were searched from inception until November, 2010 to identify relevant articles. The abstracts and titles of retrieved citations were then reviewed for relevance and potentially relevant articles were reviewed for inclusion criteria. One author (D.S.) reviewed the titles and abstracts of all potentially included articles to identify potential correlates of ADL impairment.

#### Study selection

Observational studies that identified demographic variables, measures of medical comorbidity, medical conditions, or patterns of health service utilization that were correlated with impairment in basic ADLs were eligible for inclusion. We included a condition as a potential correlate of ADL impairment provided there was at least one study identified through our review process which identified a statistically significant relationship between the potential correlate and any clinically derived measure of impairment in ADLs. We restricted our selection of studies to those that that identified variables that are routinely collected in administrative data sources which typically include diagnoses associated with medical conditions (e.g. dementia) or clinical presentations (e.g. falls). For example, we excluded correlates of functional impairment that involved measurement of variables such as laboratory values and physiological measurements such as blood pressure which are not routinely available in administrative data. Activities of daily living are classified into basic activities of daily living (e.g. ability to walk, bath oneself, toilet, and eat) and instrumental activities of daily living (e.g. shopping, managing finances or medications, and completing housework) [29]. Impairment in activities of either basic or instrumental ADLs is commonly assessed by determining the amount of assistance that an individual requires to perform a given ADL. Commonly utilized measures of basic or instrumental ADL measures often provide summary scores of the degree of assistance required in component ADLs [30]. The final list of variables correlated with ADL impairment were summarized in a table along with the range of statistical association reported between that variable and ADL impairment (e.g. odds ratios or relative risks).

### Definitions of medical conditions associated with functional impairment in administrative data

Methods for measuring each of the variables correlated with ADL impairment were identified using linked administrative healthcare databases situated at the Institute for Clinical Evaluative Sciences (ICES). These data sets were held securely in a linked, deidentified form and analysed at ICES. These datasets included the Registered Persons Database (RPDB) for demographic variables and date of death, the Canadian Institute for Health Information (CIHI) National Ambulatory Care Reporting System (NACRS) and Discharge Abstract Database (DAD) for emergency department visits and hospital admissions, respectively; the Ontario Health Insurance Plan (OHIP) claims database for outpatient and inpatient claims and assessments; and the Ontario Drug Benefits (ODB) database to identify claims for outpatient prescription medications; and the Ontario Mental Health Reporting System (OMHRS) for mental health inpatient admissions. These databases are routinely used for research purposes and the accuracy of these databases have been detailed in previous reports [31,32].

Our study population included all adults aged 66 or older who underwent hip fracture surgery (admission diagnosis: ICD-9: 820, or ICD-10: S72) in Ontario between April 1, 2003 and March 31, 2009. The correlates of functional impairment were determined using hospital discharge diagnostic codes, diagnostic codes associated with outpatient physician visits, and prescribed medications (Additional file 2). Age was categorized as 66 – 75 years, 76–85 years, and 86 years or older. The number of outpatient physician visits in the year preceding hip fracture was categorized into 10 or fewer visits, 11 – 20 visits, or greater than 20 visits. Several measures of comorbidity were measured in the study sample. The Charlson Comorbidity Index is a commonly utilized comorbidity measure [33,34] which has been adapted for use with administrative healthcare data [20,35]. The Johns Hopkins Adjusted Clinical Groups classification to measure underlying medical severity [36]. In this system, individual medical conditions are assigned to categories referred to as Aggregated Diagnosis Groups (ADGs) [22]. Eight ADGs are considered major ADGs and the sum of major ADGs determined from hospital admissions, emergency room visits or outpatient physician contacts can be utilized as a measure of comorbidity to predict outcomes [37]. We also used the sum of the number of unique medications prescribed in the year preceding index as another measure of medical comorbidity [23]. This study was approved by the ethics review board at Sunnybrook Health Sciences Centre.

### Correlates of functional impairment in study sample

We selected older adults with hip fractures as our study population because hip fractures are common among older adults [38] and functional impairment has been demonstrated to be a predictor of mortality in this population [39,40]. Hip fractures are particularly common among frail older adults who receive home care or long-term care services [41]. To determine functional impairment in our study sample, we used information contained in the Resident Assessment Instrument (RAI) derived from assessments completed for individuals who had either received home care services or who were in long-term care (LTC) prior to their hip fracture. We only included those individuals in the final study sample who had either a RAI assessment associated with home care services [42] (RAI-HC) or a RAI assessment associated with long-term care Minimum Dataset (RAI-MDS) [43] within the 90 days preceding hospital admission for hip fracture. The RAI-MDS is completed at the time of admission and quarterly for residents in LTC, the RAI-HC is completed every 6 months for individuals receiving home care services. Within both RAI instruments are several measures of ADL function. Seven components of ADL function (dressing, personal hygiene, toilet use, locomotion, transfers, bed mobility, and eating) are used to create a composite measure of ADL function referred to as the ADL long-form [44]. Each ADL item is scored from 0 (independent) to 4 (total dependence) to derive the total ADL score (range 0 – 28, higher scores indicating greater ADL impairment). The ADL long-form total score was categorized as: 5 or less; 6 – 10; 11 – 15; 16 – 20; or greater than 20. The RAI-HC [42] and RAI-MDS [45] are both accurate and reliable instruments for the measurement of demographic and clinical information in populations of older adults and are routinely used for research purposes in Ontario [46,47].

To determine whether medical conditions or other measures in administrative data were correlated with functional impairment, we performed a series of linear regression analyses with ADL long-form total scores as the dependent variable and each potential correlate as the independent variable. Variables associated with ADL impairment with a p-value of <0.05 were then retained for future models and the proportion of the variance of ADL impairment explained by the variable was assessed by the value of the R^2^ statistic. Finally, all variables that were significant in the bivariate analyses were combined in a multivariable model predicting ADL impairment, with the proportion explained variance for the model assessed by the adjusted R^2^ statistic. SAS version 9.3 (SAS Institute, Cary, North Carolina) was utilized for all analyses.

### Effect of ADL impairment on postoperative mortality

We selected 360-day mortality following hip fracture surgery as death is common in the year following hip fracture surgery with approximately 30% of older adults experiencing this outcome [48]. Follow-up data for mortality were available until March 31, 2010, therefore all individuals in the study cohort contributed a minimum of 360 days of follow-up data in which to determine mortality. Information on mortality in our databases are highly accurate with error rates of 1 – 2% [49]. The probability of mortality in the 360 days following surgery was compared across the categories of ADL impairment using a χ^2^ statistic. Then, a multivariable logistic regression model was constructed to predict the probability of 360-day postoperative mortality. We first added age, sex, and residence in LTC in sequence to the model. Next, the measures of medical comorbidity and health service utilization that were significantly correlated with ADL impairment in the study sample were added to the model. Finally, all the medical conditions that were correlated with ADL impairment were included in the model. Direct measures of ADL impairment were then added to model using dummy variables for the previously defined categories of ADL impairment. The predictive ability of the model was assessed by the c-statistic and the maximum rescaled R^2^ values obtained for the models with and without ADL impairment. To assess whether ADL impairment improved the model fit, the change in the -2 log likelihood values for the models with and without ADL impairment were compared using a χ^2^ test for with a degrees of freedom equal to the number of additional variables in the models with ADL impairment compared to the models without ADL impairment. The AIC value for the models with and without ADL impairment were also compared as another method to assess improvement in model fit with a reduction in AIC indicating improved model fit. In all of our models, we ensured that the number of events (i.e. number of individuals who died within 360 days) were sufficient for the number of covariates in order to avoid over-fitting the model using a minimum of 10 events for each covariate included [50]. There were a total of 487 individuals who died within the 360 days following hip fracture surgery, therefore, our multivariable models could include up to 48 independent variables.

## Results

### Identification of correlates of ADL impairment

A total of 195 potentially relevant articles were identified from the electronic database search, 25 articles were identified through reference lists and 4 review articles were identified [1,26,28,51]. A total of 37 articles reported on medical conditions or patient characteristics associated with basic ADL impairment (Additional file 3).

A total of 23 conditions were reported to be associated with impairment in basic ADLs (Additional file 2). These variables included demographic factors, general measures of medical comorbidity, general medical conditions, psychiatric conditions and conditions generally seen in older adult populations such as sensory impairment, falls, and incontinence. Cerebrovascular disease, cognitive impairment, and depression were the conditions most commonly reported to be associated with ADL impairment.

Information from physician outpatient visits, emergency room visits, hospital admissions and prescription drugs were identified to correspond to the medical conditions correlated with functional impairment. A medical condition was considered to be present if there was any outpatient physician visit code, emergency room, or hospitalization associated with the condition in the 5 year preceding cohort entry unless otherwise specified (Additional file 2).

### Description of hip fracture study sample

A total of 1,329 individuals were included in the final study sample of older adults with hip fractures who had either a RAI-HC assessment (N = 286) or a RAI-MDS assessment (N = 1,043) in the 90 days preceding hospitalization for hip fracture surgery (Table 1). The mean age of the sample was 85.2 years (standard deviation (SD) = 6.9) and the majority were female (72.8%). Most of the study sample resided in LTC prior to hip fracture. The study population was in frequent contact with physicians and had a high number of emergency room visits and hospital admissions in the year preceding hip fracture. There was a high prevalence of several common chronic medical problems such as dementia (79%), ischemic heart disease (40%), and osteoarthritis (43%). Overall, medical comorbidity was common in the group as reflected by the 92% of individuals having a Charlson Comorbidity Index score of 1 or more and 98% of individuals having 1 or more major ADGs. Polypharmacy was also common in this group, with the average study participant prescribed a mean of 12.3 (SD = 6.2) unique medications in the year preceding cohort entry. Impairment in ADLs prior to hip fracture was substantial in the study sample with the mean ADL score of 11.9 (SD = 7.7) and a median ADL score of 12 (interquartile range = 5–18).

**Table 1:** Baseline characteristics of study sample of older adults with hip fractures with resident assessment instrument in the 90 days preceding hip fracture

**Variable**	**Total N = 1,329**
**Demographics**	
**Age**	
Mean age, (SD)	85.2 (6.9)
66 – 75 years, N (%)	117 (8.8)
76 – 85 years, N (%)	546 (41.1)
> 85 years, N (%)	666 (50.1)
**Female sex**, N (%)	968 (72.8)
**Long-term care resident**, N (%)	1043 (78.5)
**Medical comorbidity**	
**Charlson score**, N (%)	
0	103 (7.8)
1	548 (41.2)
2	288 (21.67)
3+	390 (29.34)
**Number of major ADGs**, N (%)	
0	23 (1.73)
1	140 (10.53)
2	306 (23.02)
3	344 (25.88)
4	272 (20.47)
5+	244 (18.36)
**Number of unique drugs**, Mean (SD)	12.27 (6.18)
0 – 5 drugs, N (%)	157 (11.81)
6 – 10 drugs, N (%)	389 (29.27)
11 – 15 drugs, N (%)	430 (32.36)
>15 drugs, N (%)	353 (26.56)
**Number of outpatient visits**, Mean (SD)	26.78 (19.78)
≤ 10 visits, N (%)	184 (13.84)
11 – 20 visits, N (%)	495 (37.25)
> 20 visits, N (%)	650 (48.91)
**Number of hospitalizations**, Mean (SD)	0.41 (0.75)
Any hospital visit, N (%)	396 (29.8)
**Number of ED visits**, Mean (SD)	1.34 (1.53)
0 visits, N (%)	463 (34.84)
1 visit, N (%)	393 (29.57)
2 visits, N (%)	250 (18.81)
3 visits, N (%)	223 (16.78)
**Medical conditions**, N (%):	
Congestive heart failure	309 (23.25)
Chronic obstructive lung disease	386 (29.04)
Dementia	1057 (79.53)
Depression	804 (60.50)
Diabetes	357 (26.86)
Hearing impairment	137 (10.31)
Ischemic heart disease	540 (40.63)
Malnutrition	18 (1.35)
Any ED visit for falls	274 (20.6)
Any hospitalization for falls	24 (1.8)
Obesity	35 (2.63)
Osteoarthritis	577 (43.42)
Osteoporosis	260 (19.56)
Parkinson’s disease	74 (5.57)
Pressure ulcer	65 (4.89)
Stroke	340 (25.58)
Urinary incontinence	273 (20.54)
Visual impairment	34 (2.56)
**ADL Impairment**	
Mean ADL score (SD)	11.94 (7.66)
Median ADL score (IQR)	12 (5–18)
Range	0 - 28
ADL score category, N (%):	
0 – 5	333 (25.06)
6 – 10	268 (20.17)
11 – 16	269 (20.24)
16 – 20	248 (18.66)
> 20	211 (15.88)

ADG = Aggregated Diagnosis Groups; ADL = activity of daily living; ED = emergency department; IQR = interquartile range; N = number; SD = standard deviation.

### Correlates of functional impairment in older adults with hip fractures

In univariate linear regression analyses, several individual medical conditions or measures of medical comorbidity were associated with ADL impairment (Table 2). Variables associated with ADL impairment (p < 0.05) included residence in LTC, Charlson score (categorical), number of major ADGs (categorical), number of physician outpatient visits (continuous), congestive heart failure, COPD, dementia, hearing impairment, ischemic heart disease, number of emergency room visits for falls, osteoarthritis, Parkinson’s disease, stroke and pressure ulcers. The two variables that individually accounted for the greatest proportion of ADL impairment as assessed by the R^2^ statistic were residence in LTC (R^2^ = 0.11) and dementia (R^2^ = 0.05), while the other variables were associated with smaller R^2^ values.

**Table 2:** Correlates of impairment in basic activities of daily living among older adults with hip fractures

**Variable**	**Parameter estimate**	**Standard error**	**Test statistic**	**P value**	**R**^**2**^
**Demographics**					
Age					
Continuous	-0.0064	0.031	0.21	0.81	0.000
Categorical			F = 0.34	0.72	0.0005
66 – 75	--	--	--	--	--
76 – 85	0.46	0.78	0.59	0.53	
> 85 years	0.14	0.77	0.19	0.85	
**Sex** (Female vs Male)	0.93	0.47	1.77	0.077	0.0023
**LTC resident** (Yes vs No)	6.30	0.48	13.08	<0.0001	0.11
**Medical comorbidity**					
**Charlson score**					
Total score	0.078	0.11	0.70	0.48	0.0004
Categorical			F = 11.00	<0.0001	0.032
0	---	---	---	---	
1	5.30	0.81	6.54	<0.0001	
2	4.27	0.87	4.92	<0.0001	
3	5.09	0.97	5.28	<0.0001	
4+	4.45	0.89	5.00	<0.0001	
**Number of major ADGs**					
Total number	0.21	0.14	0.15	0.14	0.0016
			F = 2.33	0.004	0.04
Categorical					
0	----	----	----	----	
1	2.19	1.72	1.28	0.20	
2	3.09	1.65	1.87	0.06	
3	3.95	1.65	2.40	0.017	
4	3.83	1.66	2.31	0.021	
5+	2.97	1.59	1.78	0.75	
**Number of outpatient visits in past year**					
Total number	0.041	0.011	3.91	<0.0001	0.011
Categorical			F = 5.67		0.0085
≤ 10 visits	--	--	--	0.0035	
11 – 20 visits	1.51	0.66	2.28	--	
> 20 visits	2.13	0.67	3.35	0.0227	
				0.008	
**Number of unique drugs**					
Total number	-0.00016	0.034	0.00	0.99	0.000
Categorical			F = 1.63	0.18	0.0037
0 – 5 drugs	---	---	----	----	
6 – 10 drugs	1.25	0.72	1.72	0.085	
11 – 15 drugs	1.29	0.71	1.80	0.072	
>15 drugs	0.56	0.73	0.72	0.47	
Number of hospitalizations					
Total number	-0.023	0.28	-0.08	0.94	0.000
Any visit	0.025	0.46	0.53	0.59	0.0002
**Number of ED visits**	0.017	0.14	0.12	0.90	0.000
Categorical			F = 0.91	0.43	0.002
0 visits	----	-----	----	----	----
1 visit	0.57	0.53	1.08	0.28	
2 visits	-0.17	0.60	-0.27	0.78	
>2 visits	0.72	0.62	1.15	0.25	
**Medical conditions**					
CHF	-1.36	0.50	-2.75	0.0061	0.0057
COPD	-1.41	0.46	-3.07	0.0022	0.0071
Dementia	4.56	0.51	9.00	<0.0001	0.058
Depression	-0.43	0.43	-1.00	0.32	0.0008
Diabetes	-0.30	0.47	-0.65	0.52	0.0003
Hearing impairment	-1.15	0.69	-2.19	0.029	0.0036
Ischemic heart disease	-1.03	0.43	-2.40	0.016	0.0043
Malnutrition	1.57	1.82	0.87	0.39	0.0006
Number of ED visit for falls	0.99	0.34	2.95	0.0033	0.0065
Obesity	-0.24	1.31	-0.18	0.86	0.0000
Osteoarthritis	-1.19	0.42	-2.82	0.0049	0.0059
Parkinson’s disease	2.51	0.91	2.75	0.0060	0.0057
Pressure ulcers	2.03	0.97	2.08	0.037	0.0033
Stroke	1.31	0.48	2.73	0.0064	0.0048
Urinary incontinence	-0.20	0.52	-0.39	0.69	0.0001
Visual impairment	-2.27	1.33	-1.71	0.088	0.0022

ADGs = Aggregated Diagnosis Groups; ED = emergency department; LTC = long-term care.

A multivariable linear regression model was then constructed using the variables that were associated with ADL impairment in the preceding analysis. Age and sex were first entered into the model and accounted for a small proportion of ADL impairment (adjusted R^2^ = 0.005). Addition of LTC residence increased R^2^ value to 0.12. Further addition of Charlson score, number of major ADGs, and outpatient visits had a minimal effect on the adjusted R^2^ = 0.13. Addition of medical conditions associated with ADL impairment also only had a small effect on the overall adjusted R^2^ value, which was 0.16 for the final model (Table 3).

**Table 3:** Multivariable model of correlates of impairment in basic activities of daily living

**Variable**	**Parameter estimate**	**Standard error**	**Test statistic**	**P value**	**Adjusted R**^**2**^
**Age**	---	---	---	---	-0.0010
**Age, sex**	---	---	---	---	0.0005
**Age, sex, LTC**	---	---	---	---	0.1161
**Age, sex, LTC, Charlson**	---	---	---	---	0.1259
**Age, sex, LTC, Charlson, ADGs**	---	---	---	---	0.1268
**Age, sex, LTC, Charlson, ADGs, visits**	---	---	---	---	0.1311
**Age, sex, LTC, Charlson, ADGs, visits, medical conditions**					0.1687
**Age**					
66 – 75 years	0.01957	0.72	0.03	0.98	
76 – 85 years	0.24	0.42	0.57	0.57	
≥ 86 years	--	--	--	--	
**Sex**	0.63	0.44	1.44	0.15	
**LTC resident**	5.18	0.51	10.22	<0.0001	
**Charlson comorbidity score**	-1.63	1.01	-1.60	0.11	
0	-0.37	0.63	-0.59	0.56	
1	-0.73	0.66	-1.12	0.27	
2	0.76	0.74	1.02	0.31	
3	---	---	---	---	
≥ 4					
**Number of major ADGs**	1.00	1.65	0.61	0.54	
0	0.76	0.86	0.89	0.37	
1	1.03	0.69	1.50	0.13	
2	1.77	0.63	2.83	0.005	
3	0.96	0.63	1.52	0.13	
4	---	---	---	---	
≥ 5					
**Total number of outpatient visits**	0.03	0.01	3.51	0.005	
**Medical conditions**					
Congestive heart failure	-0.90	0.51	-1.77	0.07	
Chronic obstructive lung	-1.50	0.44	-3.38	0.0007	
Disease					
Dementia	2.09	0.63	3.51	0.001	
Hearing impairment	-1.07	0.64	-1.68	0.09	
Ischemic heart disease	-0.60	0.43	-1.38	0.17	
Osteoarthritis	-0.80	0.40	-1.99	0.05	
Parkinson’s disease	2.45	0.86	2.85	0.005	
Pressure ulcers	1.86	0.90	2.06	0.04	
Stroke	1.20	0.47	2.56	0.01	
Number of ER visits for falls	0.68	0.31	2.17	0.03	

ADG = Aggregated Diagnosis Groups; LTC = long-term care.

### Effects of ADL impairment, medical comorbidity, and medical conditions on postoperative mortality

Overall, 487 individuals (36.6%) died within the 360 days of hip fracture. The severity of ADL impairment was positively correlated with mortality. Individuals with an ADL score of between 0 – 5 had a mortality risk of 25.8% when compared to a risk of mortality of 43.6% for individuals with an ADL score of > 20 with intermediate categories of ADL impairment associated with a gradation in the risk of mortality (Table 4).

**Table 4:** Association between ADL impairment and mortality within 360 days of hip fracture

**ADL score**	**Died within 360 days**	**Alive at 360 days**	**Total**
	**N (%)**	**N (%)**	
**0 – 5**	86 (25.8)	247 (74.2)	333
**6 – 10**	94 (35.1)	174 (64.9)	268
**11 – 15**	111 (41.3)	158 (58.7)	269
**16 – 20**	104 (41.9)	144 (58.1)	248
**> 20**	92 (43.6)	119 (56.4)	211
**Total**	487	842	1329

ADL = activity of daily living; N = number.

χ^2^ = 26.93, d.f. 4, p = <0.0001 for difference in mortality rate associated with ADL impairment.

Impairment in ADLs was strongly associated with mortality in a logistic regression model (OR = 2.22; 95% CI: 1.54 – 3.23; χ^2^ = 26.46, P <0.0001 for highest vs. lowest level of ADL impairment). However, this logistic regression model with only ADL impairment had a weak discriminative ability (c-statistic of 0.578) and only explained a low proportion of the variation in mortality (maximum rescaled R^2^ = 0.028) (Table 5). A multivariable logistic regression model including age (categorical), sex, LTC residence, Charlson score (categorical), number of major ADGs (categorical), total number of outpatient visits (continuous), and medical conditions identified as being correlated with ADL impairment (including congestive heart failure, COPD, dementia, hearing impairment, ischemic heart disease, number of emergency room visits for falls, osteoarthritis, Parkinson’s disease, stroke and pressure ulcers) was then created. The performance of this model to predict 360 day mortality was modest with a c-statistic of 0.653 and an R^2^ value of 0.09. The addition of ADL impairment to this model resulted in statistically significant improvement in the predictive ability fit of the model according to the difference in the – 2 log likelihoods for the models with and without ADL impairment. The p-value associated with the change in – 2 log likelihoods was p = 0.002 for ADL impairment as a categorical variable and p = 0.0002 for ADL impairment as a continuous variable. This improvement in model fit was also evident with a reduction in the AIC value from 1706.94 to 1695.95 with the inclusion of ADL impairment in the model as well as improvements in the c-statistic (0.668 vs. 0.653) and maximum rescaled R^2^ (0.11 vs. 0.09).

**Table 5:** Multivariable model of predictors of mortality within 360 days of hip fracture surgery

**Variable**	**Wald chi square**	**D.F**	**P value**	**C statistic**	**Max rescaled R**^**2**^
ADL score^a^	26.46	4	<0.0001	0.578	0.0282
ADL score^b^	21.79	1	<0.0001	0.578	0.0226
Age	9.42	2	0.008	0.544	0.01
Age, sex	28.1	3	<0.0001	0.580	0.03
Age, sex, LTC	37.00	4	<0.0001	0.597	0.04
Age, sex, LTC, Charlson	53.45	8	<0.0001	0.624	0.06
Age, sex, LTC, Charlson, ADGs	60.69	13	<0.0001	0.635	0.07
Age, sex, LTC, Charlson, ADGs, visits	60.9	14	<0.0001	0.634	0.07
Age, sex, LTC, Charlson, ADGs, visits, medical conditions	81.03	24	<0.0001	0.653	0.09
Age, sex, LTC, Charlson, ADGs, visits, medical conditions, ADL impairment^a^	94.50	28	<0.0001	0.668	0.11
Age, sex, LTC, Charlson, ADG, visits, medical conditions, ADL impairment^b^	92.13	25	<0.0001	0.67	0.10

ADL = activity of daily living; ADG = aggregated diagnosis groups; LTC = long-term care.

a = ADL impairment as categorical variable, b = ADL impairment as continuous variable.

We also completed a sensitivity analysis to identify correlates of ADL impairment using backward selection in two models using P-values 0.1 and 0.05 to identify correlates. After identifying variables correlated with ADL using these models, the addition of direct measures of ADL impairment to the final model predicting mortality resulted in improved predictive ability of the model as determined changes in the AIC, -2 log likelihood ratio and c-statistic similar to the primary analysis.

## Discussion

In the current study, a number of medical conditions that are routinely recorded in administrative data and other variables were identified as being correlated with functional impairment in older adults who underwent hip fracture surgery. Impairment in basic ADLs was significantly associated with an increased risk of mortality in the year following hip fracture with a 2-fold increase in the risk of death for individuals with greatest ADL impairment as compared to those with the least amount of impairment. Importantly, even after controlling for medical comorbidity and medical conditions correlated with ADL impairment, direct measures of ADL impairment added significantly to the prediction of postoperative mortality. This suggests that measures to control for the effects of ADL impairment through use of conditions associated with functional impairment and medical comorbidity may not be sufficient when direct measures of ADL impairment are not available potentially resulting in confounding or bias in observational studies.

These findings are consistent with previous research demonstrating that ADL impairment is a significant predictor of mortality and other poor health outcomes for populations of older adults with hip fractures [39,40]. Several of the correlates of ADL impairment, such as residence in LTC, number of outpatient visits, measures of comorbidity such as the Charlson score, number of major ADGs and some medical conditions were correlated with ADL impairment in bivariate comparisons. However, in multivariable regression only residence in LTC, and certain medical conditions were correlated with ADL impairment. The association between residence in LTC and ADL impairment is predictable given that some degree of ADL impairment is required to be eligible for LTC admission in Ontario [52]. The association between dementia and ADL impairment is also anticipated given that the progressive nature of dementia results in ADL impairment especially as the disease reaches the moderate to severe stages [53]. Many of the medical conditions which were positively correlated with ADL impairment were associated with musculoskeletal dysfunction such as Parkinson’s disease and falls. These conditions, all of which have known relationships to impairment in mobility which would be expected to be reflected within our measure of ADL function. Pressure ulcers were also correlated with ADL impairment which is in keeping with immobility being the major risk factor for the development of pressure ulcers [54,55]. Counter-intuitively, the presence of some conditions such as osteoarthritis, COPD and ischemic heart disease were significantly associated with decreased likelihood of ADL impairment. One potential reason for this observation likely related to how diagnoses are recorded in administrative data. Some chronic health conditions may appear to be protective against health outcomes when they actually may be markers of individuals being in relatively good health when compared to other individuals in the same population who have other unstable health conditions which become the focus of clinical encounters by physicians and are billed as such [56].

When the outcome of mortality in the 360 days following hip fracture surgery was examined, ADL function was significantly associated with mortality in a dose response relationship. Impairment in ADLs has been demonstrated to be a predictor of postoperative mortality in older adults with hip fractures, along with several other risk factors [39,40]. While ADL impairment was a statistically significant predictor of postoperative mortality, the overall predictive value of ADL impairment was modest when considered in isolation. Addition of measures of ADL functioning to commonly used methods of risk adjustment in studies of older adults resulted in significant improvements in the predictive ability of this model although much of the variation in postoperative mortality was still unexplained by the model suggesting that some other prognostic variables were not captured in the model.

Prior studies have also demonstrated that studies utilizing administrative datasets with direct measures of functional status can significantly add to statistical models predicting outcomes such as death, institutionalization, or hospitalization [19]. A study of 6,465 participants enrolled in a cluster randomized controlled clinical trial examined the effects of usual methods of case-mix adjustment including measures of demographic variables, comorbidity scores, and measures of health service utilization to predict death, institutionalization or hospitalization. Addition of ADL measures to these models improved the predictive ability of the models although the effects of ADL measures on the overall predictive ability of the models were modest as measured by changes in the rescaled R^2^ and c-statistics. The importance of ADL impairment in predicting outcomes after adjusting for medical comorbidity has also been observed in observational studies of older adults undergoing cardiac surgery [24] and community-dwelling older adults [17]. Likewise, a modest improvement in the ability of our model to predict mortality following hip fracture surgery was also observed with the inclusion of ADL measures although the clinical significance of this is questionable.

There are some limitations to the current study. The first relates to the study population, the majority of which were in LTC prior to cohort entry along with community-dwelling older adults who received home care services. Given the high rates of functional impairment in these populations, the results observed in our study may not generalize well to other study populations with less severe levels of impairment such as community-dwelling older adults who are not receiving home care services. Some clinical variables associated with ADL impairment such as abnormal laboratory values or measures of strength were not available in our datasets and as such these could not be included in our statistical models. Finally, some conditions such as malnutrition are likely underreported in our administrative databases when compared to detailed chart review or clinical examination [57] and our methods for identifying these conditions in administrative data may also be subject to measurement error. The underreporting of some of the conditions that were identified as potential correlates of ADL impairment may have resulted in an overestimation of the relative contribution of direct measures of ADL impairment to predicting postoperative outcomes.

## Conclusions

In summary, impairment in basic activities of daily living can be an important predictor of outcomes in observational studies using administrative databases. There is the potential for residual confounding due to ADL impairment when direct measures of ADL functioning are not available, even after controlling for demographics, medical comorbidity, and medical conditions correlated with ADL impairment. Additional studies of populations other than older adults with hip fractures will be useful to further understand the impact of ADL impairment on outcomes in observational studies using administrative healthcare datasets.

## Competing interests

The authors of this manuscript have no conflicts of interest to declare.

## Author contribution

All authors made substantial contributions to the conception and design of the study. DS contributed to the analysis of data. DS drafted the manuscript and all authors revised it critically for content. The final manuscript was approved by all authors.

## Pre-publication history

The pre-publication history for this paper can be accessed here:

http://www.biomedcentral.com/1471-2318/14/9/prepub

## Supplementary Material

Additional file 1 Search terms used in search strategy. Click here for file

Additional file 2 Correlates of functional impairment in older adults. Click here for file

Additional file 3 Reference list for conditions associated with impairment in activities of daily living. Click here for file
