# Supplementary material for: Effects of impairment in activities of daily living on predicting mortality following hip fracture surgery in studies using administrative healthcare databases
Source: BMC Geriatr. 2014 Jan 28;14:9. doi: 10.1186/1471-2318-14-9 (PMC3922692; doi:10.1186/1471-2318-14-9)
Supplement: Additional file 1 — Search terms used in search strategy. [file 1471-2318-14-9-S1.docx]

**Additional file 1**: Search Terms Used in Search Strategy

MEDLINE (November 24, 2010)

1. “Activities of Daily Living”/

2. “functional impairment”.mp

3. ADL.mp

4. disability.mp

5. activities of daily living.mp

6. limitation in activity.mp

7. disablement.mp

8. functional decline.mp

9. Disability evaluation/

10. Health Status Indicators/

11. Geriatric Assessment/

12. Disabled Persons/

13. Frail Elderly/

14. frailty.mp

15. Aged/

16. “Aged, 80 and over”/

17. Age Factors/

18. Geriatrics/

19. Age Distribution/

20. Longitudinal Studies/

21. Probability/

22. Risk Factors/

23. Prevalence/

24. Cross-Sectional Studies/

25. Prognosis/

26. Cohort Studies/

27. Prospective Studies/

28. Follow-Up Studies/

EMBASE (November 24, 2010)

1. “Activities of Daily Living”/

2. “functional impairment”.mp

3. ADL.mp

4. disability.mp

5. activities of daily living.mp

6. limitation in activity.mp

7. disablement.mp

8. functional decline.mp

9. Disability evaluation/

10. Health Status Indicators/

11. Geriatric Assessment/

12. Disabled Persons/

13. Frail Elderly/

14. frailty.mp

15. Aged/

16. “Aged, 80 and over”/

17. Age Factors/

18. Geriatrics/

19. Age Distribution/

20. Longitudinal Studies/

21. Probability/

22. Risk Factors/

23. Prevalence/

24. Cross-Sectional Studies/

25. Prognosis/

26. Cohort Studies/

27. Prospective Studies/

28. Follow-Up Studies/
