# Supplementary material for: Effects of impairment in activities of daily living on predicting mortality following hip fracture surgery in studies using administrative healthcare databases
Source: BMC Geriatr. 2014 Jan 28;14:9. doi: 10.1186/1471-2318-14-9 (PMC3922692; doi:10.1186/1471-2318-14-9)
Supplement: Additional file 2 — Correlates of functional impairment in older adults. [file 1471-2318-14-9-S2.docx]

**Additional file 2**: Correlates of Functional Impairment in Older Adults

| **Condition** | **Risk Ratio for Association with ADL Impairment*** | **Measurement of Condition in Administrative Databases** | **Reference**  (see Supplementary Digital Content 3 for reference list) |
| --- | --- | --- | --- |
| ***Demographics*** |  |  |  |
| Age | 1.3 – 3.5 | RPDB | [^1-10^](#_ENREF_1) |
| Female Sex | 1.1 - 2.3 | RPDB | [^4^](#_ENREF_4)^,^[^11^](#_ENREF_11) |
| ***Measures of Medical Comorbidity*** |  |  |  |
| Number of Diseases | 1.3 |  | [^4^](#_ENREF_4)^,^[^12^](#_ENREF_12) |
| Number of Medications | 1.3 - 2.1 | Number of unique drugs in ODB in 1 year preceding index | [^5^](#_ENREF_5)^,^[^12^](#_ENREF_12)^,^[^13^](#_ENREF_13) |
| Comorbidity Score | 1.9 – 2.2 | Charlson Comorbidity Score  Major ADGs from ACG system | [^5^](#_ENREF_5)^,^[^14^](#_ENREF_14)^,^[^15^](#_ENREF_15) |
| ***Medical Conditions*** |  |  |  |
| Cerebrovascular Disease | 1.7 – 6.7 | OHIP: 432, 436  CIHI ICD- 9: 430-432, 434,  CIHI ICD- 10: I60, I61, I63, -I64 | [^3^](#_ENREF_3)^,^[^5^](#_ENREF_5)^,^[^6^](#_ENREF_6)^,^[^16-18^](#_ENREF_16) |
| Parkinson’s disease | 2.9 - 5.6 | Two diagnostic codes for Parkinson’s disease in either OHIP or CIHI within one year (excluding OHIP records occurring during hospitalizations) two prescriptions for antiparkinson medications, at least one of the script and one of the diagnostic codes must be within 2 years of each other; prescriptions must be consecutive, defined as the second being within 180 days of the first.  OHIP: 332;  CIHI ICD-9: 332.0;  CIHI ICD-10: G20.0  ODB: Parkinsons’ disease drug | [^1^](#_ENREF_1)^,^[^18^](#_ENREF_18) |
| Diabetes | 1.3 – 2.1 | Defined through diagnostic algorithm in ODD | [^2^](#_ENREF_2)^,^[^3^](#_ENREF_3)^,^[^19^](#_ENREF_19) |
| Congestive Heart Failure | 1.7 | Congestive Heart Failure, one hospitalization or two ER visits/outpatient visits within 2 years with OHIP claim  CIHI: ICD-9:   428  CIHI: ICD-10:  I500, I501, I509  OHIP: 428 | [^3^](#_ENREF_3) |
| Coronary Artery Disease | 2.7 | OHIP: 410, 412, 413;  CIHI ICD-9: 410.X , 411.X, 413.X, 414.X  CIHI ICD 10: I20.X; I21.X; I25.2 | [^17^](#_ENREF_17)^,^[^18^](#_ENREF_18) |
| Arthritis | 1.5 | OHIP: 715  ICD-9: 715.X  ICD-10: M15.0, M15.4, M15.8, M15.9 | [^6^](#_ENREF_6)^,^[^17^](#_ENREF_17) |
| Osteoporosis | -- | OHIP: 733  ICD-9: 733.0  ICD-10: M81, M82  Or receipt of any bisphophonate prior to hip fracture | [^17^](#_ENREF_17) |
| Respiratory Disease | -- | OHIP: 491, 492, 496  CIHI ICD-9: 491, 492, 496  CIHI ICD-10: J41, J42, J43, J44 | [^20^](#_ENREF_20) |
| Obesity | 2.2 | OHIP: 278  ICD- 9: 278.0  ICD-10: E66.X | [^18^](#_ENREF_18) |
| Hypertension | 2.2  -- | Hypertension  Inclusion in HYPERTENSION database | [^17^](#_ENREF_17)^,^[^18^](#_ENREF_18) |
| ***Psychiatric Conditions*** |  |  |  |
| Psychiatric Disorder | 5.9 |  | [^18^](#_ENREF_18) |
| Cognitive Impairment | 1.2 - 3.6 | OHIP: 290, 331, 797  CIHI ICD- 9: 290.0, 290.1, 290.3, 290.4, 290.8, 290.9, 294.1, 294.8, 294.9, 331.0, 331.1, 331.2, 797.0  CIHI ICD-10: F00.0, F00.1, F00.2, F00.9, F01.0, F01.1, F01.2, F01.3, F01.8, F01.9, F02.0, F02.1, F02.2, F02.3, F02.4, F02.8, F03.X, F05.1, F06.5, F06.6, F06.8, F06.9, F09.X, G300.0, G30.1, G30.8, G30.9, G31.0 G31.1, R54.X | [^1^](#_ENREF_1)^,^[^5^](#_ENREF_5)^,^[^7^](#_ENREF_7)^,^[^8^](#_ENREF_8)^,^[^10^](#_ENREF_10)^,^[^11^](#_ENREF_11)^,^[^14^](#_ENREF_14)^,^[^18-29^](#_ENREF_18) [^12^](#_ENREF_12)^,^[^16^](#_ENREF_16)^,^[^17^](#_ENREF_17)^,^[^30^](#_ENREF_30) |
| Depression | 1.2 – 2.1 | Depression: either any inpatient admission for depression or at least one outpatient visit to GP or psychiatrist with depression code AND one antidepressant prescription   - CIHI ICD-9 296.2, 296.3; 300.4; 311 - CIHI ICD-10 F32, F33, F412, F480 - OMHRS: (Axis I diagnosis): 296.2X, 296.3X, 296.9, 311.X - OHIP: 300, 311 - Prescription of antidepressant medication | [^11-13^](#_ENREF_11)^,^[^17^](#_ENREF_17)^,^[^19-22^](#_ENREF_19)^,^[^31-35^](#_ENREF_31) |
| ***Geriatric Conditions*** |  |  |  |
| Visual Impairment | 1.6 – 1.7 | OHIP: 369  CIHI ICD-9: 369.00, 369.01, 369.04, 369.08, 369.10, 369.20, 369.22, 369.3  CIHI ICD-10: H54.0, H54.2, H54.3 | [^8^](#_ENREF_8)^,^[^12^](#_ENREF_12)^,^[^13^](#_ENREF_13)^,^[^16^](#_ENREF_16)^,^[^19^](#_ENREF_19)^,^[^32^](#_ENREF_32)^,^[^36^](#_ENREF_36) |
| Hearing Impairment | -- | OHIP: 389  CIHI ICD-9: 389  CIHI ICD-10: H90.0, H90.3, H90.6 | [^12^](#_ENREF_12)^,^[^32^](#_ENREF_32) |
| Malnutrition | 1.3 | OHIP: 263  CIHI ICD-9: 262, 263.0, 263.1, 263.8, 263,9,  CIHI ICD-10: E43, E44.0, E44.1 | [^11^](#_ENREF_11) |
| Urinary Incontinence | 1.8 – 1.9 | OHIP: 625, 788;  CIHI ICD- 9: 625.6, 788.3, 788.30, 788.31, 788.32, 788.33, 788.34, 788.37, 788.39;  CIHI ICD-10: N39.3, R32.X, N39.4  ODB claim for urinary incontinence drug | [^1^](#_ENREF_1)^,^[^26^](#_ENREF_26)^,^[^37^](#_ENREF_37) |
| Pressure Ulcer | 3.2  -- | OHIP: 707;  CIHI ICD-9: 707.00, 707.02, 707.03, 707.04, 707.05, 707.06, 707.07, 707.09;  CIHI ICD-10: L89 | [^1^](#_ENREF_1)^,^[^9^](#_ENREF_9) |
| Falls | 1.2 – 2.3 | Any ER visit or hospitalization with: any S or T00-T14 (injury to single or multiple sites) with W00-W19 (falls as external site of injury) | [^1^](#_ENREF_1)^,^[^2^](#_ENREF_2)^,^[^5^](#_ENREF_5)^,^[^13^](#_ENREF_13)^,^[^20^](#_ENREF_20)^,^[^26^](#_ENREF_26) |
