# Supplementary material for: Effects of impairment in activities of daily living on predicting mortality following hip fracture surgery in studies using administrative healthcare databases
Source: BMC Geriatr. 2014 Jan 28;14:9. doi: 10.1186/1471-2318-14-9 (PMC3922692; doi:10.1186/1471-2318-14-9)
Supplement: Additional file 3 — Reference list for conditions associated with impairment in activities of daily living. [file 1471-2318-14-9-S3.docx]

**Additional file 3:** Reference List for Conditions Associated with Impairment in Activities of Daily Living

**1.** Cornette P, Swine C, Malhomme B, Gillet J-B, Meert P, D'Hoore W. Early evaluation of the risk of functional decline following hospitalization of older patients: development of a predictive tool. *Eur J Public Health.* Apr 2006;16(2):203-208.

**2.** Covinsky KE, Hilton J, Lindquist K, Dudley RA. Development and validation of an index to predict activity of daily living dependence in community-dwelling elders. *Med Care.* Feb 2006;44(2):149-157.

**3.** Gill TM, Allore HG, Holford TR, Guo Z. Hospitalization, restricted activity, and the development of disability among older persons. *Jama.* Nov 3 2004;292(17):2115-2124.

**4.** Mor V, Wilcox V, Rakowski W, Hiris J. Functional transitions among the elderly: patterns, predictors, and related hospital use. *Am J Public Health.* Aug 1994;84(8):1274-1280.

**5.** Volpato S, Onder G, Cavalieri M, et al. Characteristics of nondisabled older patients developing new disability associated with medical illnesses and hospitalization. *J Gen Intern Med.* May 2007;22(5):668-674.

**6.** Boult C, Kane RL, Louis TA, Boult L, McCaffrey D. Chronic conditions that lead to functional limitation in the elderly. *Journal of gerontology.* Jan 1994;49(1):M28-36.

**7.** Sager MA, Rudberg MA, Jalaluddin M, et al. Hospital admission risk profile (HARP): identifying older patients at risk for functional decline following acute medical illness and hospitalization. *J Am Geriatr Soc.* Mar 1996;44(3):251-257.

**8.** Tas U, Verhagen AP, Bierma-Zeinstra SMA, Odding E, Koes BW. Prognostic factors of disability in older people: a systematic review. *British Journal of General Practice.* Apr 2007;57(537):319-323.

**9.** Miller EA, Weissert WG. Predicting Elderly People's Risk for Nursing Home Placement, Hospitalization, Functional Impairment, and Mortality: A Synthesis. *Med Care Res Rev.* 2000;57:259-297.

**10.** McCusker J, Kakuma R, Abrahamowicz M. Predictors of Functional Decline in Hospitalized Elderly Patients: A Systematic Review. *J Gerontol.* 2002;57A:M569-M577.

**11.** Chen CC-H, Dai Y-T, Yen C-J, Huang G-H, Wang C. Shared risk factors for distinct geriatric syndromes in older Taiwanese inpatients. *Nurs Res.* Sep-Oct 2010;59(5):340-347.

**12.** Landi F, Liperoti R, Russo A, et al. Disability, more than multimorbidity, was predictive of mortality among older persons aged 80 years and older. *J Clin Epidemiol.* Jul 2010;63(7):752-759.

**13.** Stuck AE, Walthert JM, Nikolaus T, et al. Risk factors for functional status decline in community-living elderly people: a systematic literature review. *Soc Sci Med.* Feb 1999;48(4):445-469.

**14.** Stuck AE, Walthert JM, Nikolaus T, Bula CJ, Hohmann C, Beck JC. Risk factors for functional decline in community-living elderly people: a systematic literature review. *Social Science & Medicine.* 1999;48:445-469.

**15.** Lang PO, Meyer N, Heitz D, et al. Loss of independence in Katz's ADL ability in connection with an acute hospitalization: Early clinical markers in French older people. *Eur J Epidemiol.* Sep 2007;22 (9):621-630.

**16.** Formiga F, Ferrer A, x00E, et al. Risk factors for functional decline in nonagenarians: a one-year follow-up. The NonaSantfeliu study. *Gerontology.* 2007;53(4):211-217.

**17.** Wang L, van Belle G, Kukull WB, Larson EB. Predictors of functional change: a longitudinal study of nondemented people aged 65 and older. *J Am Geriatr Soc.* Sep 2002;50(9):1525-1534.

**18.** Wolff JL, Boult C, Boyd C, Anderson G. Newly reported chronic conditions and onset of functional dependency. *J Am Geriatr Soc.* May 2005;53(5):851-855.

**19.** Dunlop DD, Semanik P, Song J, Manheim LM, Shih V, Chang RW. Risk factors for functional decline in older adults with arthritis. *Arthritis Rheum.* Apr 2005;52(4):1274-1282.

**20.** Miller EA, Weissert WG. Predicting elderly people's risk for nursing home placement, hospitalization, functional impairment, and mortality: a synthesis. *Medical Care Research & Review.* Sep 2000;57(3):259-297.

**21.** Li LW, Conwell Y. Effects of changes in depressive symptoms and cognitive functioning on physical disability in home care elders. *J Gerontol A Biol Sci Med Sci.* Feb 2009;64(2):230-236.

**22.** Mehta KM, Yaffe K, Covinsky KE. Cognitive impairment, depressive symptoms, and functional decline in older people. *J Am Geriatr Soc.* Jun 2002;50(6):1045-1050.

**23.** Rothman MD, Leo-Summers L, Gill TM. Prognostic significance of potential frailty criteria. *J Am Geriatr Soc.* Dec 2008;56(12):2211-2116.

**24.** Moritz DJ, Kasl SV, Berkman LF. Cognitive functioning and the incidence of limitations in activities of daily living in an elderly community sample. *American Journal of Epidemiology.* Jan 1 1995;141(1):41-49.

**25.** Gill TM, Williams CS, Richardson ED, Tinetti ME. Impairments in physical performance and cognitive status as predisposing factors for functional dependence among nondisabled older persons. *Journals of Gerontology Series A-Biological Sciences & Medical Sciences.* Nov 1996;51(6):M283-288.

**26.** Cigolle CT, Langa KM, Kabeto MU, Tian Z, Blaum CS. Geriatric conditions and disability: the Health and Retirement Study. *Annals of Internal Medicine.* Aug 7 2007;147(3):156-164.

**27.** Blaum CS, Ofstedal MB, Liang J. Low cognitive performance, comorbid disease, and task-specific disability: findings from a nationally representative survey. *Journals of Gerontology Series A-Biological Sciences & Medical Sciences.* Aug 2002;57(8):M523-531.

**28.** Sands LP, Xu H, Craig BA, Eng C, Covinsky KE. Predicting change in functional status over quarterly intervals for older adults enrolled in the PACE community-based long-term care program. *Aging Clin Exp Res.* Oct 2008;20(5):419-427.

**29.** Boaz RF. Improved versus deteriorated physical functioning among long-term disabled elderly. *Med Care.* Jun 1994;32(6):588-602.

**30.** Nikolova R, Demers L, x00E, land F, ois. Trajectories of cognitive decline and functional status in the frail older adults. *Arch Gerontol Geriatr.* Jan-Feb 2009;48(1):28-34.

**31.** Yang Y, George LK. Functional disability, disability transitions, and depressive symptoms in late life. *J Aging Health.* Jun 2005;17(3):263-292.

**32.** Tinetti ME, Allore H, Araujo KLB, Seeman T. Modifiable impairments predict progressive disability among older persons. *J Aging Health.* Apr 2005;17(2):239-256.

**33.** Dorantes-Mendoza G, Avila-Funes JA, Mejia-Arango S, Gutierrez-Robledo LM. Factors associated with functional dependence in older adults: A secondary analysis of the National Study on Health and Aging, Mexico, 2001. [Spanish]. *Revista Panamericana de Salud Publica/Pan American Journal of Public Health.* July 2007;22 (1):1-11.

**34.** Ballard C, Hanney ML, Theodoulou M, et al. The dementia antipsychotic withdrawal trial (DART-AD): long-term follow-up of a randomised placebo-controlled trial. *Lancet Neurology.* Feb 2009;8(2):151-157.

**35.** Penninx BWJH, Geerlings SW, Deeg DJH, van Eijk JTM, Tilburg W, Beekman ATF. Minor and Major Depression and Risk of Death in Older Persons. *Archives of General Psychiatry.* 1999;56:889-895.

**36.** Lee JSW, Chau PPH, Hui E, Chan F, Woo J. Survival prediction in nursing home residents using the Minimum Data Set subscales: ADL Self-Performance Hierarchy, Cognitive Performance and the Changes in Health, End-stage disease and Symptoms and Signs scales. *Eur J Public Health.* Jun 2009;19(3):308-312.

**37.** Coll-Planas L, Denkinger MD, Nikolaus T. Relationship of urinary incontinence and late-life disability: implications for clinical work and research in geriatrics. *Z Gerontol Geriatr.* Aug 2008;41(4):283-290.
